# On-Farm Welfare Assessment Protocol for Suckling Piglets: A Pilot Study

**DOI:** 10.3390/ani10061016

**Published:** 2020-06-10

**Authors:** Marika Vitali, Elena Santacroce, Federico Correa, Chiara Salvarani, Francesca Paola Maramotti, Barbara Padalino, Paolo Trevisi

**Affiliations:** Department of Agricultural and Agri-food Sciences and Technologies, University of Bologna, Viale Fanin 46, 40138 Bologna, Italy; marika.vitali4@unibo.it (M.V.); elena.santacroce@studio.unibo.it (E.S.); federico.correa2@unibo.it (F.C.); chiara.salvarani4@unibo.it (C.S.); francesca.maramotti@studio.unibo.it (F.P.M.); barbara.padalino@unibo.it (B.P.)

**Keywords:** animal welfare, swine, aggressive behaviour, tail biting, suckling piglets, skin lesions, ear biting, housing conditions, tail-docking, tear staining

## Abstract

**Simple Summary:**

The welfare of piglets is a major concern for the pig industry. Despite a large body of knowledge regarding piglets welfare, only a few specific protocols to assess welfare have been proposed for suckling piglets. Consequently, there is limited implementation of monitoring of piglets welfare during the nursery phase. Therefore, the present study tested the ability of a new protocol regarding the identification of the main welfare issues in suckling piglets and their relationship to management conditions. This pilot study involved 134 litters from two farms, with a total of 1608 piglets assessed at the age of 7 and 20 days. In both farms, some litters were tail docked, while others were left undocked. The welfare parameters consisted of behavioural, lesion and health measures. The results showed that the main issues were represented by lesions in the front area of the body, probably the consequence of teat competition due to repeated cross-fostering, and lack of appropriate milk supplementation. Non-aggressive lesions and health conditions were mainly related to housing conditions such as light and pen and nest temperatures. Tail docking did not influence lesions or behaviour; however, tail-docked piglets showed high scores in the indicators of a negative emotional state.

**Abstract:**

Piglets experience welfare issues during the nursery phase. This pilot study aimed to test a protocol for identifying the main welfare issues in suckling piglets and to investigate relationships among animal-based indicators and management conditions. Litters (n = 134), composed of undocked and tail-docked piglets, were assessed at two farms. After birth, observations were made at the age of 7 days and 20 days. At each observation, housing conditions (HCs) were measured, and 13 animal-based indicators, modified from Welfare Quality, Classyfarm, Assurewel and others introduced ex novo, were recorded. A generalized linear mixed model was used, considering animal-based indicators as dependent variables and farm, piglets’ age, tail docking and HCs as independent variables. The main welfare issues were lesions of the limb (32.6%) and the front area of the body (22.8%), a poor body condition score (BCS) (16.1%), ear lesions (15.5%), and tail lesions (9.7%). Negative social behaviour (e.g., fighting and biting) represented 7.0% of the active behaviour, with tail biting observed in 8.7% of the piglets. While lesions on the front areas of the body were mostly associated with the farm, tail lesions, low BCS, tear staining, and diarrhoea were associated with light and nest temperature (*p* < 0.05). In particular, tail biting increased with scarce light (*p* = 0.007). Tail docking did not influence any animal-based indicator except for tear staining which was higher in the tail-docked as compared to the undocked piglets (*p* = 0.05), increasing awareness on this practice as a source of negative emotion in piglets. The protocol tested may be a promising tool for assessing on-farm piglets’ welfare.

## 1. Introduction

The nursery phase has been considered to be one of the most challenging phases in swine production [1,2]. Especially in intensive rearing systems, and when hyper-prolific genetics are used, piglets are exposed to many risks regarding animal welfare, e.g., mutilation, a high mortality rate within the first 24 h, hypothermia, and high teat competition [3,4]. Currently, aggressive behaviour and tail biting, resulting in skin and tail lesions, are considered major welfare issues in all phases of pig production [5,6]. The effort on reducing skin and tail lesions is usually directed towards post-weaning piglets [7,8]; however, there is evidence that those lesions could also develop during the suckling period [9]. Consequently, it has been suggested that prevention strategies for minimising these lesions should start soon after birth [7,8]. 

Many housing conditions and management procedures have been linked to the welfare issue and with the manifestation of aggressive behaviour [10]. For example, when hyperprolific sows are employed, the management of large litter size has been reported to affect piglets welfare since they can increase the competition for resources [11]. Teat competition could increase the frequency of facial lesions starting at one week of age in suckling piglets [12]. With inadequate cross-fostering, fighting and competition will also increase, resulting in lesions on the front area of the body and impairing productive parameters [5,13]. Management strategies, such as providing supplementary milk to piglets, have been studied from a productive point of view [11,14]; however, their effect on aggressive behaviour has not yet been investigated. Housing conditions such as ambient temperature [2] and light intensity [15,16] might also influence suckling piglets behaviour, even if their relationship with aggressive behaviour and lesion outcomes is unclear. Performing tail docking is still one of the methods routinely used by farmers in the many EU Member States to reduce tail biting behaviour [17], even though there is consolidated scientific knowledge reporting that tail docking is not efficient in minimising tail biting [18]. It is very invasive and has been proven to affect piglets’ welfare and behaviour in the subsequent weeks [19,20]. Moreover, it is banned by Dir. 120/2008 EC. Prevention strategies have been proposed [21,22] and, of them, a decrease in space allowance, appropriate environmental enrichment and a good human-animal relationship have been shown to reduce the occurrence of tail lesions in growing and finishing pigs [23,24]. Despite that, the identification of effective measures capable of reducing as well as preventing tail lesion has been reported to be critical by the farmers [25]. It is likely that, since tail biting has a multifactorial basis, to identify which factors trigger this behaviour is uneasy, and because of that, it is still a matter of debate [7]. 

The development of a protocol for scientific risk assessment regarding aggressive behaviour and tail biting is highly recommended by the Racc 366/2016 EC, but no specific on-farm protocols for suckling piglets have been found. The first step in developing a risk assessment protocol is to develop a method for welfare assessment in piglets using animal-based measures [26]. The use of animal-based measures is presently considered to be the most reliable in assessing animal welfare at any age since the measures are taken directly from the animal. Measuring behaviour and lesions on the body or the observation of clinical signs are examples of animal-based measures. According to the opinion of the European Food Safety Authority (EFSA), the association of scientific evidence between animal-based criteria and management factors is a pivotal step in the improvement of animal welfare in livestock animals [26]. 

The present study hypothesised that there would be significant associations between management conditions and piglets’ welfare during the nursing phase. In the present study, a list of animal-based indicators of negative and positive welfare status was tested on two farms. The first aim of the study was to quantify and qualify the main welfare issues of suckling piglets using a new combination of animal-based criteria. The second aim was to investigate the possible associations of the welfare indicators with tail docking, piglets’ age, farms, and housing conditions. 

## 2. Materials and Methods 

### 2.1. Ethical Statement

Experimental procedures, involving piglets reared under conventional farm conditions, complied with the European Code of Practice for care and use of animals for scientific purposes (DL n. 116, 27 January 1992). Before the experiment began, the procedure was explained to the owners and written informed consent was obtained. Since no tissues or any other samples were collected, there was no need for approval by the Italian Health Ministry in agreement with EU legislation DL n. 116, 27 January 1992. 

### 2.2. Animals and Experimental Protocol

The present study involved 134 litters (average number of piglets/litter 12.0 ± 1.9) reared on two conventional Italian farms: farm 1 (F1, 73 litters) and farm 2 (F2, 61 litters), respectively. Both farms were located in the so-called “Italian Food Valley”, and the piglets were reared for the production of Parma Ham PDO (Protected Designation of Origin). The litters were randomly chosen, according to Welfare Quality protocol [27]. 

**Figure 1:**
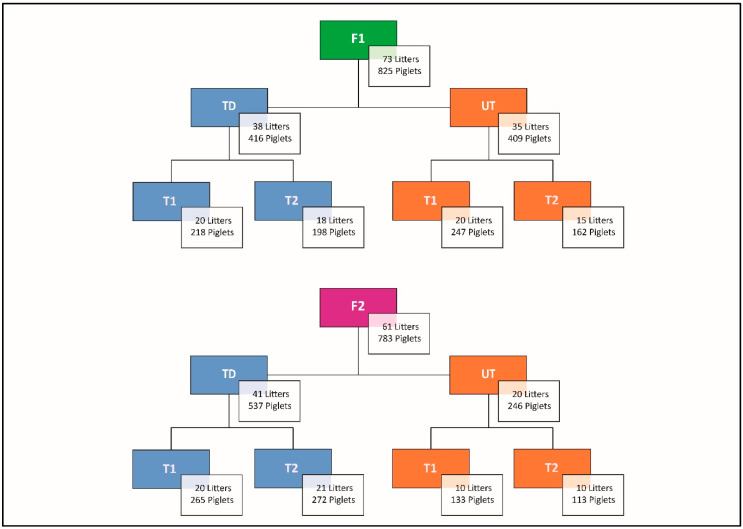
Scheme of observations. In the study, a total of 134 litters (1608 piglets) were assessed on two farms (F1, F2). The piglets on each farm were assessed on two different age (T1 = 7 days; T2 = 20 days of age). On each farm, there were litters with undocked (UT) and docked tails (TD). Tail docking was performed without local anaesthesia within the first week of life.

The experimental protocol and group composition are summarized in Figure 1. Briefly, data collection was carried out on two groups, tail docked (TD), and undocked (UT) piglets, at two piglets’ age, namely at 7 days (T1) and at 20 days, the day before weaning (T2). Briefly, data collection was carried out using a 2 × 2 × 2 factorial design; on both farms litters with tail docked (TD) and undocked (UT) piglets were assessed at two piglet ages, namely 7 days (T1) and 20 days (T2) of age. Before T1 all the males were castrated and all TD piglets were tail-docked, while T2 was the day before weaning.

### 2.3. Nutrition and Housing Condition

Before starting the data collection, face-to-face interviews with the farmers were conducted by two Authors (P.T., M.V.) using a questionnaire (File S1). The questionnaire was used to explore how each farm was managed. 

The answers to the questionnaire showed that the two farms did not differ in the number of employees/sow (1 employee/<2000 animals), neither in employee education and training (at least five years of experience or educational qualifications and periodic training courses on animal welfare). Genetically, the sows were of the hyperprolific type (Danbred^®^) and the piglets were conformed to Italian heavy pig production. Both farms used conventional farrowing crates with a nesting area constituted of full-floor under a warm lamp. Farrowing was performed weekly in both farms and cross-fostering was performed many times by both farmers. Ventilation was mixed. F1 performed tail docking and castration on piglets at the age of 3 days, while F2 performed them at 6 days. Both farms did not use anaesthetic or analgesic during or after the mutilation procedures. None of the farms performed teeth clipping. Weaning took place at 21 days in both farms. Starting at three days of life, the piglets were fed supplementary milk in addition to sow’s milk. On F1, supplementary milk was available ad libitum in a drinking cup placed in each farrowing crate. On F2, supplementary milk was provided twice a day, in a round trough placed inside the farrowing crate. Both farms provided water to piglets during the entire nursery phase, by means of a nipple drinker placed on the corner of the farrowing crate. Neither of the farms used any enrichment materials for the piglets. To assess housing conditions (HCs) between the two farms, on each assessment day, differently trained people (E.S., F.C., C.S., F.P.M.) recorded HCs in each litter under the supervision of an expert evaluator (M.V.). Light intensity and pen temperature were recorded at the piglets’ level considering three points in the pen: the corner closest to the centre of the room, in the middle and the opposite corner closer to the external wall and an average of the three measures was calculated. Nest temperature was measured only in one point, and corresponded to the middle position under the lamp. Light intensity was measured using a Mini Light Meter (UNI-T UT383, Dongguan City, China), whereas temperature was recorded using a Datalogger (UNI-T UT330C USB, Dongguan City, China). The area of the farrowing crate was calculated using a Laser Distance Meter (Extech DT40M, Nashua, NH, USA) and was then divided by the number of piglets, i.e. space allowance. Analyses of variance (ANOVA) revealed no differences between the two farms (F1, F2), two age groups (T1, T2), and tail docking status (TD, UT) in terms of HCs. The HCs are expressed as the mean of all the measures and standard deviation (SD). The piglets were kept at an average space allowance of 0.3 (± 0.1) m^2^ with an average litter size of 12.0 (±1.9) piglets. Light intensity was 23.7 (±28.3) lux (measured at the piglets’ eye level). The pen temperature was 26.1 (±2.9) °C and the nest temperature 29.0 (±2.8) °C. On the contrary, light intensity, pen, and nest temperatures presented high variability among the litters; they were therefore included in the analysis to identify their relationship to animal-based parameters.

### 2.4. Welfare Parameters

The welfare parameters were modified from Welfare Quality [26], Classyfarm [28], and AssureWel [29] protocols, with some introduced ex novo after literature review. Full references and an explanation of the parameters are reported in Table 1.

Briefly, the parameters were divided into qualitative behaviour assessment (QBA), behavioural measurements (BMs), lesions, and health measurements (LHMs). The QBA, LHMs, and BMs were always assessed by the same person (M.V.), with 5-years of expertise on welfare assessment in piglets and trained on how to apply the Welfare Quality and Classyfarm protocols. 

The QBA was carried out between 9:00–10:00 am outside the pen and consisted of four observation time points (5 min each) for a total of 20 min for each experimental group (Figure 1). Data were reported on a 125 mm scale and multiplied for the coefficients indicated in the Welfare Quality protocol, to calculate the QBA score [27]. Two measures of QBA were taken per each experimental group. Higher values in the QBA score corresponded to a positive emotional state. 

The BMs were evaluated outside the pen, between 10:00 and 11:00 am by direct observation of all the piglets in each litter, three times per litter at an interval of 5 minutes each. For each behaviour, an average of the three observations was calculated. Behavioural measurement consisted of two types of observation: (i) category of behaviour as described in the Welfare Quality protocol [27]; (ii) individual or stereotyped behaviour. The category of behaviour included “social behaviour”, “exploratory behaviour”, “other active behaviours”, and “inactive behaviour”, are detailed in Table 1. The frequency of “social”, “exploratory”, and “other active behaviours” was determined on the total of active behaviour in each litter. Frequency of “inactive behaviour” was calculated on the total behaviour observed, as explained in the Welfare Quality protocol for pig [27]. Observed stereotyped and individual behaviour included negative social behaviour (such as fighting and ear, tail and body biting); and positive social behaviour (play) (Table 1). They were calculated as the percentage of the animals exhibiting the behaviour over the total of animals in the litter:

((n. of piglets demonstrating the behaviour/total of piglets in the litter)*100).

The LHMs were always assessed on each piglet of the litter in the afternoon, and the assessment was carried out inside the pen at a distance of 0.5 m from the piglet using a headlight when necessary. Skin lesions were visually scored in each piglet on a 0 to 2 scale (0 = up to 4 lesions, 1 = 5 to 10 lesions, 2 = more than 11 lesions); the percentage of each score was then calculated in each litter. The prevalence of lesions in each area was determined in each litter as a percentage of the sum of score 1 and 2 scores. The lesion score index (LSI), which considered both the frequency and the severity of the lesions, was then calculated in each area as follows (range 0 to 200, where 0 is absence and 200 all animals with severe lesions/tear staining [32]):

Area LSI = (% of lesion score 1+ (2* % of lesion score 2)).

Other LHMs were recorded on the piglet using a Y/N score (Y = presence, N= absence) and the prevalence of Y score was calculated per litter. 

### 2.5. Statistical Analysis

All the statistical analyses were carried out using R software [33]. Descriptive analyses of all welfare parameters were carried out using the psyc.ir package [34]. 

Differences in the QBA score were tested using a general linear model (GLM) procedure in the Stats package [33] with the QBA score as a dependent variable, and farm, piglets’ age, and tail docking as fixed factors. The statistical unit in the QBA score was the experimental group. Differences among QBA scores were tested using ANOVA (lsmeans package, [35]). QBA descriptors underwent to principal component analysis (PCA) using the FactoMineR package [36].

For the BMs and the LHMs, percentages below 5% did not undergo additional statistical analyses. In the case of LHMs having a 0 to 2 score, the percentage of piglets in each litters having 1 and 2 scores in a certain area were summed and only measurements having an average prevalence ≥ 5% underwent additional analysis. The association between the welfare measurement and the HCs were then evaluated using a general linear mixed model (GLMM), using the lme4 package [37]). The GLMM was carried out on BMs and LHMs using the measures as dependent variables, farm, piglets’ age and tail docking as factors, light, pen, and nest temperatures as the covariates, and litter size as a random factor. Analysis of variance was used to evaluate the differences among factors. Results in the text were presented as the mean of the value (±SD). Statistical significance was set at *p* ≤ 0.05. 

## 3. Results

### 3.1. Qualitative Behaviour Assessment

**Figure 2:**
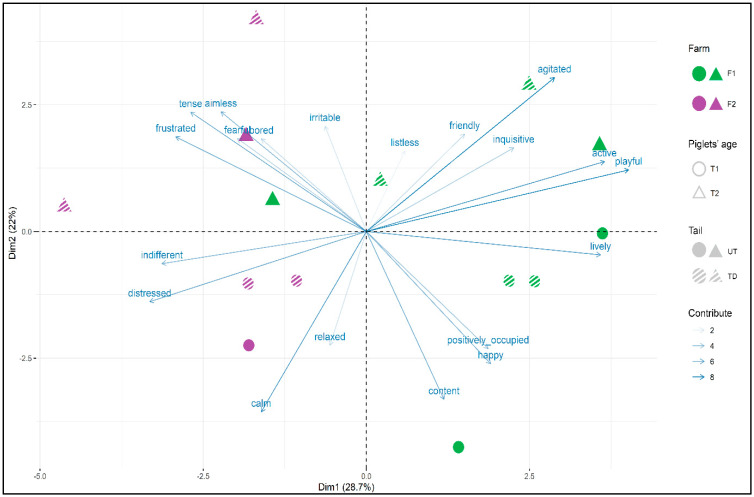
Qualitative behaviour assessment (QBA) analysis performed on the piglets. The QBA was performed following the indication in the Welfare Quality [27]. Descriptors and factors were analysed using principal component analysis. The results of Dim 1 and Dim 2 are reported. One spot corresponded to one observation. Colour of the spot indicated the farms: F1 (green) and F2 (purple). The shape of the spot corresponded to piglets’ age: T1 = piglets at the age of 7 days (circle); T2 = piglets at the age of 20 days (triangle). The texture represented the tail: UT = undocked tail (solid coloured spot); TD = tail-docked (striped spot). The arrows represent the eigenvalues of the descriptors and thickness of the arrows represent the average contribution of each descriptor on the dimensions.

On average, the QBA score was 32.1 (±24.4). The QBA score significantly differed between F1 and F2 (44.3 vs. 16.1, *p* < 0.0001), which indicated a more positive emotional state in F1. A higher score was also found in T1 compared to T2 (31.2 vs. 24.5, *p* = 0.0002), indicating a worse emotional state at the age of 20 days as compared to 7 days. Piglets who did not receive tail docking showed a higher QBA score as compared to TD piglets (31.2 vs. 24.5, *p* = 0.01). The two first components were retained from the PCA, explaining 28.7% and 22.0% of the total variation of QBA score (Figure 2, File S2). Dimension 1 (Dim 1) was characterised by descriptors of valence in the emotional state, and ranged from active and lively (eigenvalue 0.8) to distressed (eigenvalue −0.7), while Dimension 2 (Dim 2) was, for the most part, characterised by descriptors related to arousal, ranging from agitated (eigenvalue 0.7) to calm (eigenvalue −0.8), even if the cluster was less clear. The farms clustered clearly, and they differed by their loading in Dim 1. In fact, F2 piglets had a negative score on Dim 1, signifying that they were perceived as more tense, aimless, frustrated, distressed and indifferent than those on F1, on which the piglets were more active, playful, lively, happy and content. Piglets’ age clustered in Dim 2 signifying that the T2 piglets were perceived as having more high arousal (agitated, active, playful, fearful) than the T1 piglets. Tail docking did not cluster in any of the dimensions, showing that this factor did not differ in its expression of valence nor arousal. 

When the interaction between the two axes was considered, it was possible to observe that the T2 piglets from F2 were strongly associated with negativity and displeasure descriptors (negative valence and high arousal), while at the same age, the piglets in F1 were associated with the positive activity (positive valence and high arousal). 

### 3.2. Behavioural Measurements

Considering all the litters, 58.6% (±29.8) of the piglets were inactive during the assessment. Regarding active behaviour, the 45.4% (±38.1) of piglets showed other active behaviour (which, for the most part, involved only suckling or eating) or pen exploring behaviour (32.9 ± 33.0%). Social behaviour was shown only by the 9.2% (±11.1) of piglets out of all active behaviour, which consisted of negative (6.5 ± 15.2%) and positive (2.5 ± 6.9%) behaviour. The negative social behaviour observed in piglets was fighting (9.8 ± 26.9%), tail biting (7.9 ± 24.2%), and body biting (4.8 ± 17.5%, which involved nipples, penis, legs, and flank), and ear-biting (4.3 ± 17.4%). The positive social behaviour observed was play (7.2 ± 25.0%). Regarding tail position, 67.2% (±25.3) of the piglets showed the curly tail position, 22.1% (±18.9) of piglets had a hanging or low tail, and 10.5% (±13.6) had tucked low tail. Tail-docking and piglets’ age did not influence any of the behaviour tested (*p* > 0.05); F1 showed an increased hanging down tail posture as compared to F2 (*p* = 0.01) (Table 2). Analysis of variance evidenced no significant differences for the following behaviour: pen exploration, other active behaviour, and inactive behaviour (Table 2).

The effect of HCs on behaviour was reported in Table 3. Tail biting showed to be negatively influenced by light (*p* = 0.007), showing a lower occurrence of the behaviour with higher lux. The frequency of piglets showing negative social behaviour was inversely influenced by pen temperature (*p* = 0.047), thus the behaviour was more frequently observed with lower temperatures in the pen. Curly tail posture was in inverse proportion with pen temperature (*p* = 0.007) while a high frequency of hanging down tail posture resulted associated with high pen temperature (*p* = 0.007). Additionally, with low nest temperature, the frequency of piglets showing hanging down tail increases (*p* = 0.013). Tucking down tail position, on the contrary, was not affected by any of the tested parameters (*p* > 0.05).

### 3.3. Lesion and Health Measures

**Figure 3:**
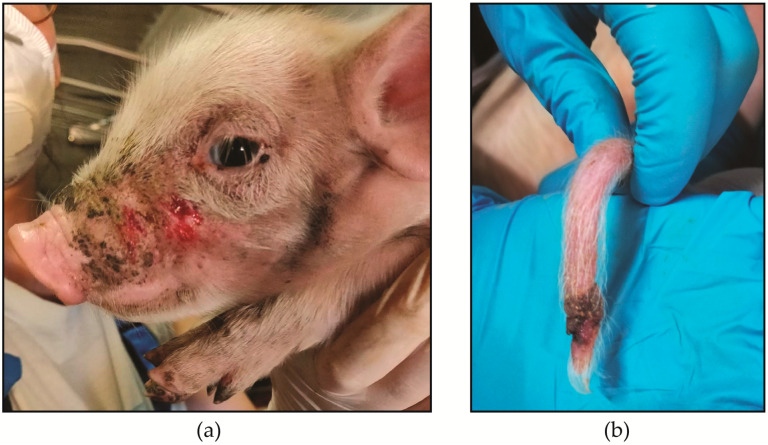
Example of severe lesions (score 2) observed in the study. (**a**) = front lesions; (**b**) = tail lesion.

The average prevalence above 5% of LHMs were as follows: limb lesions (32.7% ± 23.2), front (22.8% ± 19.7), tear staining (19.8% ± 20.9), low body condition score (16.1% ± 22.3), ear (15.5% ± 20.0), tail lesions (9.7% ± 11.9), and diarrhoea (5.2% ± 12.8). An example of the front and tail lesions emerged from the study is in Figure 3. 

A full descriptive analysis (also considering the severity score) is reported in File S3. The results of ANOVA are reported in Table 4 and Table 5.

Tail lesion score index (LSI) (which considers only lesions due to biting) was not influenced by tail docking but significantly increased with piglets’ age (*p* = 0.002).

The farms were the main factor influencing ear and front LSI, higher in F2 as compared to F1 (*p* = 0.002 in both). Tear staining LSI was affected by both farm and tail docking; it was higher on F1 as compared to F2 (*p* = 0.027) and in TD as compared to UT (*p* = 0.050). 

Housing conditions significantly influenced LHMs (Table 5). Ear LSI and diarrhoea prevalence decreased (*p* = 0.032 and *p* = 0.001, respectively) with higher illumination. At higher pen temperatures, a higher tail LSI, a major prevalence of a low body condition score, and diarrhoea were found (*p* = 0.020, *p* = 0.020, and *p* = 0.008, respectively). Tail LSI and diarrhoea were negatively associated with nest temperature (*p* = 0.042 and *p* < 0.0001, respectively).

## 4. Discussion

Results from the pilot study showed that the protocol tested was able to identify the main welfare issues in suckling piglets, and documented that farms, piglets’ age, and HCs each had a different impact on piglets’ welfare during the nursery phase. 

The main welfare issues which where identified can be divided into three main categories: issues derived by aggressive behaviour, abnormal behaviours, and poor health conditions. It is worth noting that the skin lesions derived by aggressive behaviour usually present a comma shape (if they are the consequence of biting due to fighting or competition for the resources), and are mainly located on the front-third of the body; or long and parallel (if the consequence of mounting), mainly in the middle and back areas of the body since they are caused by the impact of the claws [38]. In the present study, lesions imputable to aggressive behaviour were located in the front areas of the body (i.e., facial and front lesions). Those types of lesions have been known to be the consequence of teat competition, as has already been shown in piglets from hyperprolific sows and/or when inadequate or repeated cross-fostering is carried out [3,4]. Piglets in each litter developed a fidelity for a certain teat in the first 24 h of life [13,39]. Repeated cross-fostering after this time will lead to competition for the same teat between the new and the old piglets [40] and fighting to re-establish the hierarchy in the litter [13]. Due to this, continuous cross-fostering, as was carried out on both farms in the present study, is not recommended by the literature [3]. Consequently, the data in the present study suggested that the management of the piglets should be improved to enhance their welfare.

Lesions derived from abnormal behaviour are mainly the result of non-aggressive biting [41]. In the present study, the piglets showed warning levels (more than 5% of the piglets showing the lesions) of ear and tail lesions, which have been reported as indicators of stress and poor welfare in pigs of all stages [42]. The BMs showed the presence of ear and tail biting behaviour in the litters. Despite the area of the body involved, those behaviours share commonalities in the underlying motivations [43,44]. Non-aggressive biting may have multifactorial origins, considering a lack of cognitive stimuli as the crucial factor in the development of those behaviours [41]. 

Behavioural measurements demonstrated that, in the “active behaviour” category, “pen exploratory behaviour” and “other behaviours” were the main active behaviours observed in the litters studied. In addition, as expected, “other behaviours” was, for the most part, the result of suckling. The higher frequency of “pen exploratory behaviour” confirmed that piglets, starting from early life, have a strong motivation to explore. Pigs explore to acquire information regarding their surroundings and relative resources [45]; however, in a barren environment, exploratory behaviour can often be considered to be a signal of stress since the farming environment did not provide appropriate stimuli [44,45]. If not correctly managed, the motivation to explore might turn into abnormal behaviour, such as stereotypies or redirected behaviour toward pen mates, resulting in lesions in many areas of the body, especially the tail, ear, flank, and vulva [46]. “Negative social behaviour” was observed in the present study and consisted mainly of fighting, tail and ear biting, and body biting (involving, for the most part, nipples, penis, legs, and flank biting). The presence of both ear and tail lesions and negative social behaviour along with exploratory behaviour emphasised the need for preventive measures in the nursery. In the present study, the farms did not provide environmental enrichment for the piglets. The present results increased awareness regarding the needs of environmental devices for piglets in the nursery since they have been found to prevent the development of abnormal behaviour and to increase positive social behaviour (i.e., playing) at this stage [47,48,49]. The presence of other stressors (such as hierarchical instability due to continuous cross-fostering), disease, inappropriate management and housing conditions could indirectly lead to an increase in non-aggressive biting behaviour [41,49].

Welfare issues not directly related to aggressive or abnormal behaviour were limb lesions, low body condition score (BCS), and diarrhoea. Limb lesions, in particular, are known to be the direct consequence of the contact of the limbs with the slatted floor in the attempt to reach the udder or when resting [50,51,52]. These lesions are often severe and open wounds, representing access for pathogens, and are a source of lameness in piglets [50]. A low BCS and diarrhoea are the main factors affecting piglets’ health and the mortality rate in the nursery phase [1,2]. The greater prevalence of piglets showing a low BCS is a common welfare issue of piglets from hyperprolific sows, even if it can be influenced by management strategies or by the health conditions of piglets or the sow [41]. Undernutrition, both pre- and post-birth, has been considered to be a stressor with possible long-term consequences on the maturation of the neuroendocrine system, with possible but no consistent effects on aggressive behaviour and tail- or ear-biting [41]. A low BCS can also be a consequence of diarrhoea resulting from bacterial and virus infections [2]. Diarrhoea is a common disease in suckling piglets, having a broad aetiology, involving the interaction between environment and management, pathogens, and host conditions [2,53]. 

This study found that there was an association between the farm and the presence of front and ear lesions with F2 showing a higher lesion score than F1. Since the front lesions were, for the most part, imputable to teat competition, the different provision of supplementary milk to piglets could have influenced the higher occurrence of front lesions in F2 since they received supplementary milk twice a day instead of continuously, as on F1. The positive effect of the automatic milk replacer on the productive parameters of piglets from hyperprolific sows has already been reported [12,14]; however, to the best of the Authors’ knowledge, no specific studies have been found regarding how reconstituted milk, provided in addition to sow milk, could affect facial lesions and behaviour. The present study did not investigate the behaviour of piglets during lactation events and, since aggression is more frequent at this stage, the Authors recommend that measuring competition during lactation should be utilised for the validation of front lesions as an indicator of competitiveness in suckling piglets. The higher score on ear lesions in F2 as compared to F1 seems to confirm that piglets’ welfare was lower in F2. Ear lesions, which were predominantly the outcome of ear-biting, are largely known as indicative of higher stress in pigs [42]. Thus, this hypothesis should be of interest for additional investigations.

Besides, the results from the QBA showed a lower QBA score for the piglets in F2, with a higher association of negative emotional state descriptors: tense, aimless, frustrated, distressed, and indifferent. On the contrary, the piglets on F1 were more associated with the descriptors active, playful, lively and content. The differences in the QBA score could also have been influenced by the age in which the piglets were tail-docked and castrated. Piglets on F1 were tail-docked and castrated 3 days before the T1 assessment, while in the piglets on F2, it was carried out the day before. This result could have contributed to the lower score on F2 as compared to F1. To the best of Authors’ knowledge, no studies tested QBA as an indicator of emotional state in piglets in the days after the mutilation procedures. Behavioural studies have reported behavioural pain indicators (vocalization, lethargy, tail wagging and rubbing the body area) in the few hours after the mutilations, but failed to see clear indicators between 6 and 48 hours later in the case of tail docking, while the indicators were visible up to 4 days for castration [54,55]. It is also likely that the age of mutilation could have affected the piglets’ emotional state. Even though no studies were found on QBA regarding this topic, Lessard et al. [56] observed higher immunosuppression in piglets castrated at the age of 10 days instead of 3 days. Changes in suckling behaviour and vocalization were also reported to increase between the age of 3 and 10 days [57], suggesting that a late procedure would have a more detrimental effect on piglets welfare. The present results indicated that the use of the QBA might be of interest for further studies as an indicator of the emotional state of piglets following different mutilation procedures.

Tear staining was inserted into the protocol as an indicator of the piglets’ emotional state [30,58]. Tear staining was assessed in the left eye, as, according to the literature, this eye is connected to the right brain hemisphere which plays a pivotal role in processing negative emotion [59]. Tear staining resulted significantly higher in F1 compared to F2. Increased tear staining was associated with increased stress in the late rearing phase, in finisher pigs [60]. The same Author also reported that tear staining was linked to tail lesions, as was also observed by Telkänranta et al. [31] in growing-finishing pigs, while Parois et al. [61] found tear staining to be associated with skin lesions in weaned piglets. However, F1 showed higher welfare and lower lesion scores and a higher positive emotional state when considering LHMs and QBA scores and descriptors. Therefore, the higher score in tear staining on F1 was not in accordance with the results of the other welfare parameters. This could suggest that other factors, not considered in the present study, could have had an impact on tear staining (e.g., dustiness, gas concentration, dirtiness) as previously suggested [60]. 

Piglets’ age influenced the tail lesion score, higher at the age of 20 days than 7 days. The tail lesion score did not differ between the two farms and was not influenced by tail docking, however, it increased at the age of 20 days compared to 7 days. Tail biting is currently considered to be an iceberg indicator of poor welfare so far, having a negative effect on the emotive state of piglets [57]. In agreement with the last statement, the QBA score was significantly lower at T2. The results of the PCA showed descriptors imputable to high arousal clustering at T2. This is not surprising, since physiologically, the piglets started to be more active at this age, and the activity level normally increases in the post-weaning phase. Despite this, no changes in active behaviour were observed in BMs. Direct behavioural observation, as in the present study, may have represented a limitation. In fact, although the behavioural measurements were carried out in the same range of time, the observations were not conducted simultaneously. Therefore, some observation of the behaviour could have been biased by the difference due to the occurrence of the nurse event; however, this avoided inter-observer variability. Video-analysis would have been a more appropriate method; however, since the protocol was intended for on-farm monitoring, the use of video recording was not always feasible due to the technology required (e.g., in the precision livestock farming), and the fact that it might be more time-consuming. 

Tail docking did not influence the occurrence of any lesion or health measures, except for the tear staining score which was, higher in the TD piglets as compared to the UT piglets. As detailed above, the presence of tear staining has recently been considered to be an indicator of a negative emotional state in pigs [62]. Accordingly, the QBA score was significantly low in TD as compared to the UT, even if the descriptors did not cluster for this parameter. It seemed to indicate the overall negative emotional impact of tail docking procedure in the TD group without any (positive or negative) association with tail lesions or tail biting in piglets at this stage. The present results were in disaccord with what has been observed by Tallet et al. [62,63] who observed no changes in tear staining but reported a reduction in tail lesions in suckling piglets undergoing tail docking as compared to undocked piglets. Similarly, Reiner et al. [64] also observed a reduction in tail lesions in docked piglets vs. undocked ones. The explanation for these differences is attributable to differences in experimental design, tail lesion assessment and above all, the multifactorial origin of the problem. In fact, when the welfare condition is good, the need for tail docking is unnecessary and, similarly, under poor welfare conditions, tail docking is not effective in preventing tail biting outbreaks [19]. 

Association were also found regarding housing conditions. The light was inversely associated with the occurrence of ear lesions and tail biting behaviour, showing a higher ear lesions score and more tail-biters piglets with scarce illumination. The effects of light intensity have been poorly investigated in pigs kept indoors. In the present study, the piglets were kept at an average light intensity of 27.1 lux, thus below the level required by Dir 128/2008 EC. The current legislation recommends a minimum of 40 lux for pigs at any age; this provision should guarantee the needs of pigs for explorative and social activities [65]. Despite this, there is still a traditional belief that keeping pigs in semi-dark conditions will reduce aggressiveness, even though this belief has no scientific confirmation and has been confirmed as a baseless practice [66]. Low illumination levels have been shown to impair pigs vision of object or conspecifics [67] and to increase cortisol levels [68]. Some studies have shown that providing more illumination has a positive effect on reducing competitive behaviour in finishing pigs [69] and feed activity in suckling piglets [16], although no studies have investigated the effect of light on tail and ear biting in suckling piglets. A greater proportion of pigs showing low BCS and diarrhoea was also negatively associated with light intensity in the present study. Reduced illumination has been found to reduce feed intake in pigs [70]. The reduced light exposition has shown also to influence leukocyte counts and plasma cortisol, leading possibly to a major susceptibility to diseases in finishing pigs [68]. Therefore, the present results recommend investigating the effect of illumination on piglet health and welfare. 

Nest temperature was negatively associated with tail lesions and diarrhoea. Accordingly, in piglets, at lower nest temperatures were associated with more hanging and tucking down tail positions. Tail position has recently been proposed as an indicator of tail biting damage in piglets; however, it has also been observed to be an indicator of an impaired health condition [71], negative emotional state, fear and pain in piglets [72,73,74,75]. 

On the contrary, pen temperature was directly associated with tail lesion score, diarrhoea, a low BCS and the hanging down tail positions. These results indicated poorer piglet welfare associated with higher pen temperatures. On average, pen temperatures were 26.1 °C, ranging from 20.7 °C to 32.2 °C. It is important to consider that, if the higher temperatures are suitable for piglets, they are deemed challenging for the sows. In the present study the health and welfare conditions of the sows were not assessed; however much has been written regarding the effect of heat stress on lactating sows. The present results pointed out that appropriate nest and pen conditions were crucial for maintaining piglets health. It should, however, be noted that adequate pen temperatures for the sows are also crucial, since, with elevated temperatures, agalactia and issues regarding the sow’s health can occur, having a detrimental impact on piglets health and welfare as well. Heat stress was reported to cause agalactia [41] and poor milking in the sow. Agalactia is recognized as one of the main factors affecting piglets body weight [2]. Piglets with a low BCS usually experience a low average daily gain and weigh less at weaning, compromising their welfare also in the post-weaning phases. High pen temperature resulted associated also with tail biting in the present study. Some studies have previously reported the contemporary presence of low BCS and tail lesions in some farms. A negative correlation between the severity of the tail lesion and weight at weaning, and between the prevalence of pigs with severe tail lesions in a herd and average daily gain in weaners were observed in a previous study [75]. In a study by Van de Weerd et al. [49], it was reported that piglets showing persistent tail biting were the smallest piglets. Beattie et al. [76] observed that biter piglets showed a lower daily gain at weaning, suggesting that nutritional deficiency during lactation might affect the occurrence of tail biting.

This pilot study had some limitations. The protocol was tested on only two farms, and therefore, it needs to be validated using a larger number of farms. Only direct observation was carried out and, although the behaviours were recorded in the same range of time, the observations were not conducted simultaneously. Therefore, some observations of the behaviour may have been biased by the differences in time between the occurrence of the nursery event and this actual observation. Moreover, the different age in which mutilation procedures are carried out on piglets should be taken into account. In addition, some indicators of positive/negative emotional state were preliminarily tested. Of the indicators, tear staining seemed to be a promising indicator although more investigation is necessary to standardise its assessment to avoid overlapping factors which may bias the results, as detailed above.

Overall, once improved, the protocol may allow an easy on-farm monitoring system, which should be applied by both veterinarians and trained technicians, and may be of support for the farmers in term of reducing aggressive and abnormal damaging behaviour and enhancing more positive welfare condition in suckling piglets.

## 5. Conclusions

This pilot study found that the protocol proposed was promising for the identification of welfare issues and their relationship with the management of suckling piglets. The main welfare issues were lesions on the limbs, front third of the body, a low BCS, the ear and tail lesions and diarrhoea, and therefore the distress that can be associated with them. Behavioural measurement indicated the extensive manifestation of the exploratory behaviour towards the pen, and only a minor part of the social negative behaviour, including tail biting that, was the commonest negative behaviour observed in piglets. This study found associations between the lesions of the anterior part of the body and the farm, and between tail- and ear-lesions, diarrhoea and low BCS and housing conditions, such as light, and pen and nest temperature. Overall, the results in the present study demonstrated the needs of improving the knowledge as to which indicators are effective as to evidence welfare status in suckling piglets and as to how housing conditions may affect piglets’ welfare. 

## Figures and Tables

**Table 1:** List of parameters measured in the study, level of sampling, references and description. The parameters were ordered according to the time-line of the study.

Type	Parameter	Level	Reference	Description
QBA ^1^	Qualitative Behaviour Assessment	Treatment group ^4^	[27]	The value was expressed in mm on a scale of 125 mm (Visual Analogue Scale for QBA).
BM ^2^	Social behaviour (negative and positive)	Litter	[27]	Modified from the reference. Negative social behaviour included any aggressive social behaviour or biting causing a response from the animal disturbed. Positive social behaviour consisted of sniffing, licking, playing and moving gently away from the other animal without an aggressive or fighting reaction from this individual. Negative and positive social behaviour were recorded, and they were expressed as the % of social behaviour (positive or negative)/ the % of total active behaviour (sum of social, exploratory and other behaviours).
BM	Exploratory behaviour (pen and environmental enrichment - directed)	Litter	[27]	Modified from the reference. Pen- and enrichment- directed exploratory behaviour were recorded, and they were expressed as the % of exploratory behaviour (pen or environmental enrichment directed)/ the % of total active behaviour (sum of social, exploratory and other behaviours).
BM	Other active behaviours	Litter	[27]	Any active behaviour not included in the previous categories.
BM	Inactive behaviour	Litter	[27]	Any behaviour when the animal remained motionless thus without any activity.
BM	Tail biting (TB)	Litter	-	The piglets were attempting to manipulate or bite the tail of a pen mate.
BM	Ear biting (EB)	Litter	-	The piglets were attempting to manipulate or bite the ear of a pen mate.
BM	Body biting (BB)	Litter	-	The piglets were attempting to manipulate or bite a part of the body of a pen mate (e.g., flank, genitals, legs etc.).
BM	Fighting (F)	Litter	-	Piglets involved in the fighting.
BM	Play (P)	Litter	-	Piglets playing with one or more pen mates.
BM	Tail position	Individual	[30]	Tail posture was classified as follows: curly tail; tail hanging down; tail tucked down (down and tucked to the body).
LHM ^3^	Skin lesions	Individual	[27]	Considers 5 separate areas (ear, front, middle, hind-quarters, legs). Score was 0 = up to 4 visible lesions; 1 = 5–10 visible lesions; 2 = 11 to 15 visible lesions.
LHM	Tail lesions	Individual	[27]	Modified from the reference. 0 = absence of lesions; 1 = superficial biting along the length of the tail but no evidence of swelling or blood; 2 = fresh blood visible on the tail, the presence of a scar, swelling, or missing a part of the tail.
LHM	Tear staining	Individual	[31]	The presence of red tears in the left eye. Modified from the reference as follows: 0 = absence of staining; 1 = staining barely detectable or less than 50% of the total eye area; 2 = staining up to 100% of the eye area or extending below the mouth.
LHM	Low body condition score	Individual	[27]	Any piglets which were very lean or too small when compared to the others in the litter.
LHM	Diarrhoea	Individual	[27]	Modified from the reference. The presence or absence of this parameter was assessed in each piglet observed individuals.
LHM	Neurological disorder	Individual	[27]	Modified from the reference. Includes muscle tremor or paddle-like limbs. In this category turned head, loss of equilibrium or any other clinical sign of a neurological disorder were also included. Presence or absence of this parameter was assessed in each observed individuals.
LHM	Hernia	Individual	[27]	Modified from the reference. The presence or absence of this parameter was assessed in each piglet observed.
LHM	Limb lesions	Individual	[27]	Modified from the reference. The presence or absence of this parameter was assessed in each piglet observed.
LHM	Lameness	Individual	[27]	Modified from the reference. The presence or absence of this parameter was assessed in each piglet observed.
LHM	Further care	Individual	[29]	Identifies animals which have to be removed from the pen, needing additional care or being emergency culled. The presence or absence of this parameter was assessed in each piglet observed.

^1^ QBA = qualitative behaviour assessment; ^2^ BM = behavioural measurement; ^3^ LHM = lesion and health measurement. ^4^ Treatment group = these groups corresponded to the 8 categories of the experiment.

**Table 2:** Effect of the farm, piglets’ age, and tail docking on the behaviour observed. The values are estimated least-square means of the value.

	Farm				Piglets’ Age				Tail-Docking			
	F1	F2	SEM *	*p*-Value	T1	T2	SEM *	*p*-Value	UT	TD	SEM *	*p*-Value
**Pen** **explorative behaviour ^1^**	39.3	20.9	0.27	0.529	22.0	37.0	0.26	0.216	22.4	36.2	0.26	0.107
**Negative social** **behaviour ^1^**	6.2	3.9	0.34	0.830	7.0	3.5	0.33	0.795	4.6	5.3	0.35	0.339
**Other active** **behaviour ^1^**	35.0	48.9	0.25	0.737	55.2	32.8	0.25	0.939	42.1	43.0	0.25	0.427
**Inactive** **behaviour ^2^**	60.3	57.4	0.08	0.648	58.6	59.2	0.08	0.922	59.2	58.6	0.08	0.936
**Tail biting ^3^**	0.1	0.0	1.46	0.569	0.0	0.0	1.42	0.196	0.0	0.1	1.44	0.250
**Curly tails ^4^**	66.7	66.0	0.05	0.187	66.7	66.0	0.05	0.879	62.8	69.5	0.05	0.177
**Hanging down tails ^4^**	20.3	18.2	0.22	**0.013**	18.5	19.9	0.22	0.645	20.7	17.8	0.22	0.730
**Tucking down tails ^4^**	8.6	11.0	0.29	0.555	9.0	10.5	0.277	0.71	20.7	17.8	0.22	0.784

* SEM = Standard error of means. ^1^ The values were calculated as the mean of the behaviour/ total active behaviour observed (%). ^2^ The values were calculated as the mean of inactive behaviour/total active behaviour observed (%). ^3^ The values were mean of the piglets showing the behaviour/ total of piglets in each litter (%).^4^ The values were means of the prevalence of piglets exhibiting the tail posture in each litter (%). Values in bold evidenced significant association (*p* < 0.05).

**Table 3:** Effect of light, pen temperature, nest temperature, on the observed behaviour in suckling piglets.

	Mean	Light					T° Pen					T° Nest				
		Effect	Estimate	se	Chi sq	*p*-Value	Effect	Estimate	se	Chi sq	*p*-Value	Effect	Estimate	se	Chi sq	*p*-Value
**Pen** **exploration ^1^**	32.5	↔	0.01	0.01	0.49	0.484	↔	0.07	0.01	1.13	0.289	↔	−0.04	0.07	0.40	0.529
**Negative social behaviour ^1^**	6.7	↔	0.01	0.35	0.86	0.354	↓	−0.18	0.01	3.93	**0.047**	↔	−0.02	0.08	0.05	0.151
**Other active behaviour ^1^**	45.4	↔	−0.01	0.01	1.23	0.268	↔	0.06	0.06	0.81	0.369	↔	−0.03	0.08	0.11	0.153
**Inactive behaviour ^2^**	56.6	↔	0.00	0.00	0.02	0.879	↔	0.01	0.02	0.05	0.829	↔	0.01	0.02	0.07	0.787
**Tail biting ^3^**	7.9	↓	−0.04	0.02	7.25	**0.007**	↔	−0.12	0.11	1.31	0.253	↔	−0.07	0.12	0.32	0.584
**Curly tails ^4^**	67.2	↔	−0.00	0.00	0.71	0.399	↓	−0.04	0.01	7.17	**0.007**	↔	−0.02	0.07	0.99	0.320
**Hanging down tails ^4^**	22.1	↔	−0.01	0.01	1.05	0.306	↑	0.16	0.06	7.29	**0.007**	↓	−0.18	0.07	6.16	**0.013**
**Tucking down tails ^4^**	10.5	↔	-0.00	0.01	0.199	0.656	↔	0.08	0.07	1.190	0.275	↓	−0.15	0.07	4.29	**0.038**

↓ = negative effect; ↑ = positive effect; ↔ = no effect. ^1^ The values were calculated as the mean of the behaviour/ total active behaviour observed (%). ^2^ The values were calculated as the mean of inactive behaviour/total active behaviours observed (%). ^3^ The values were mean of the piglets showing the behaviour/ total of piglets in each litter (%).^4^ The values were means of the frequency of piglets’ exhibiting the tail posture in each litter (%). Values in bold evidenced significant association (*p* < 0.05).

**Table 4:** Effect of the farm, piglets’ age and tail docking on lesions and health parameters. The numbers are estimate least-square means of the value.

	Farm				Piglets’Age				Tail Docking			
	F1	F2	SEM *	*p*-Value	T1	T2	SEM *	*p*-Value	UT	TD	SEM *	*p*-Value
**Ear LSI ^1^**	9.3	32.1	0.28	**0.002**	12.8	23.1	0.27	0.132	22.2	13.3	0.27	0.194
**Front LSI ^1^**	19.1	48.9	0.21	**0.002**	24.5	38.1	0.20	0.126	30.9	30.3	0.21	0.946
**Leg LSI ^1^**	5.5	7.0	0.32	0.600	5.3	7.4	0.35	0.537	8.1	4.8	0.34	0.325
**Tail LSI ^1^**	7.0	13.5	0.27	0.107	5.4	17.6	0.26	**0.002**	12.9	7.3	0.28	0.187
**Tear staining LSI ^1^**	28.5	12.6	0.25	**0.027**	14.6	24.5	0.24	0.120	13.2	27.1	0.25	**0.050**
**Low BCS ^2^**	10.4	18.5	0.29	0.177	11.9	16.1	0.28	0.457	14.9	12.9	0.28	0.735
**Diarrhoea ^2^**	3.3	2.5	0.42	0.561	3.3	2.5	0.42	0.677	2.7	3.2	0.35	0.728

* SEM = Standard error of means. ^1^ The lesion score index (LSI) is calculated on a range of 0–200 considering the prevalence and severity of the lesions or tear staining in the are considered, where 0 is absence and 200 is all animals with severe lesions/tear staining. ^2^ The scores were calculated as the prevalence of piglets showing the presence of the clinical sign. Values in bold evidenced significant association (*p* < 0.05).

**Table 5:** The effect of light, pen temperature, and nest temperature on lesion and health parameters in suckling piglets. The numbers are least-square means of the value.

	Mean	Light					T° Pen					T° Nest				
		Effect	Estimate	se	Chi sq	*p*-Value	Effect	Estimate	se	Chi sq	*p*-Value	Effect	Estimate	Se	Chi sq	*p*-Value
**Ear LSI ^1^**	19.6	↓	−0.02	0.01	4.58	**0.032**	↔	−0.03	0.08	0.14	0.714	↔	−0.06	0.06	0.97	0.325
**Front LSI ^1^**	32.9	↔	0.00	0.00	0.00	0.999	↔	0.00	0.05	0.90	0.344	↔	−0.01	0.04	0.02	0.895
**Leg LSI ^1^**	6.6	↔	−0.00	0.01	0.01	0.917	↔	−0.14	0.10	1.88	0.170	↔	−0.12	0.11	1.18	0.278
**Tail LSI ^1^**	12.1	↔	0.00	0.01	0.00	0.956	↑	0.12	0.07	5.37	**0.020**	↓	−0.14	0.07	9.93	**0.042**
**Tear staining LSI ^1^**	23.5	↔	0.01	0.01	0.77	0.382	↔	0.10	0.06	2.25	0.133	↔	0.01	0.07	0.02	0.901
**Low BCS ^2^**	16.1	↔	0.00	0.01	0.02	0.877	↑	0.16	0.07	5.40	**0.020**	↔	0.03	0.10	0.06	0.804
**Diarrhoea ^2^**	5.2	↓	−0.04	0.01	11.60	**0.001**	↑	0.24	0.09	6.99	**0.008**	↓	−0.62	0.53	19.30	**<0.0001**

↓ = negative effect; ↑ = positive effect: ↔ = no effect. ^1^ The LSI is calculated on a range of 0-200 considering the prevalence and severity of the lesions or tear staining in the area considered, where 0 is absence and 200 is all animals with severe lesions/tear staining. ^2^ The scores were calculated as the prevalence of piglets showing the presence of the clinical sign (%). Values in bold evidenced significant association (*p* < 0.05).

## Supplementary Materials

The following are available online at https://www.mdpi.com/2076-2615/10/6/1016/s1, File S1: Farm questionnaire, File S2: Qualitative behaviour assessment, File S3: Descriptive analysis.
