# Supplementary material for: On-Farm Welfare Assessment Protocol for Suckling Piglets: A Pilot Study
Source: Animals (Basel). 2020 Jun 10;10(6):1016. doi: 10.3390/ani10061016 (PMC7341312; doi:10.3390/ani10061016)
Supplement: Supplementary file 1 [file animals-10-01016-s001.zip › Supplementary file_rev1/File S1-Farm questionnaire.docx]

**Date:**

**Farm:**

**Breeding stage:** Nursery (pre-weaning)

**Number of sows:**

**Farrowing schedule:**

**Farrowing crates and floor type:**

**Breed and/or genetics:**

**Presence of pigs with undocked tail:**

| N. | notes |
| --- | --- |
|  |  |
|  |  |

**Number of employees**

- **0**= 1 employee / < 2000 animals;
- **1**= 1 employee / 2000-4000 animals;
- **2**= 1 employee / > 4000 animals

**Education level of owner and employees**

- **0**= at least five years of experience or educational qualifications and periodic training courses on animal welfare and practical instructions to all employees;
- **1**= at least five years of experience or educational qualifications and training courses on animal welfare in the last three years and practical instructions to all employees;
- **2**= less than five years of experience and no educational qualifications and/or no welfare training course or not even a single employee presence without practical instructions received.

**Procedures in case of tail biting outbreak:**

- **0=** presence of written procedures and the employers were trained;
- **1**= no written procedure buy the employee were trained;
- **2=** absence of both

**Emergency culling procedure:**

- **0=** presence of written procedures and the employees were trained;
- **1**= no written procedure buy the employees were trained;
- **2=** absence of both

**Feeding:**

**Water source:**

**Castration:**

| Castration |  |
| --- | --- |
| Piglets age |  |
| Type of castration |  |
| Use of anesthesia |  |
| Use of analgesic |  |

**Teeth reduction:**

**Ventilation:**

- Natural
- Artificial
- Mixed

**Notes:**
